# Evaluation of risk equations for prediction of short-term coronary heart disease events in patients with long-standing type 2 diabetes: the Translating Research into Action for Diabetes (TRIAD) study

**DOI:** 10.1186/1472-6823-12-12

**Published:** 2012-07-09

**Authors:** Shou-En Lu, Gloria L Beckles, Jesse C Crosson, Dorian Bilik, Andrew J Karter, Robert B Gerzoff, Yong Lin, Sonja V Ross, Laura N McEwen, Beth E Waitzfelder, David Marrero, Norman Lasser, Arleen F Brown

**Affiliations:** 1 School of Public Health, University of Medicine and Dentistry of New Jersey, 683 Hoes Lane West, RM 220, Piscataway, NJ, 08854, USA; 2 Centers for Disease Control and Prevention, Atlanta, GA, USA; 3 Department of Family Medicine and Community Health – Research Division, UMDNJ-Robert Wood Johnson Medical School, Somerset, NJ, USA; 4 Department of Internal Medicine/Metabolism, Endocrinology and Diabetes, University of Michigan, Ann Arbor, MI, USA; 5 Division of Research, Kaiser Permanente, Oakland, CA, USA; 6 Pacific Health Research Institute, Honolulu, HI, USA; 7 Kaiser Center for Health Research Hawaii, Honolulu, HI, USA; 8 Department of Medicine, Indiana University, School of Medicine, Indianapolis, IN, USA; 9 Department of Medicine, University of Medicine and Dentistry of New Jersey, New Jersey Medical School, Newark, NJ, USA; 10 Division of General Internal Medicine and Health Services Research, University of California, Los Angeles, School of Medicine, Los Angeles, CA, USA

## Abstract

**Background:**

To evaluate the U.K. Prospective Diabetes Study (UKPDS) and Framingham risk equations for predicting short-term risk of coronary heart disease (CHD) events among adults with long-standing type 2 diabetes, including those with and without preexisting CHD.

**Methods:**

Prospective cohort of U.S. managed care enrollees aged ≥ 18 years and mean diabetes duration of more than 10 years, participating in the Translating Research into Action for Diabetes (TRIAD) study, was followed for the first occurrence of CHD events from 2000 to 2003. The UKPDS and Framingham risk equations were evaluated for discriminating power and calibration.

**Results:**

A total of 8303 TRIAD participants, were identified to evaluate the UKPDS (n = 5914, 120 events), Framingham-initial (n = 5914, 218 events) and Framingham-secondary (n = 2389, 374 events) risk equations, according to their prior CHD history. All of these equations exhibited low discriminating power with Harrell’s c-index <0.65. All except the Framingham-initial equation for women and the Framingham-secondary equation for men had low levels of calibration. After adjsusting for the average values of predictors and event rates in the TRIAD population, the calibration of these equations greatly improved.

**Conclusions:**

The UKPDS and Framingham risk equations may be inappropriate for predicting the short-term risk of CHD events in patients with long-standing type 2 diabetes, partly due to changes in medications used by patients with diabetes and other improvements in clinical care since the Frmaingham and UKPDS studies were conducted. Refinement of these equations to reflect contemporary CHD profiles, diagnostics and therapies are needed to provide reliable risk estimates to inform effective treatment.

## Background

Adults with diabetes have an increased risk of coronary heart disease (CHD) [1,2]. Their risks of having an initial CHD event and the prediction equations used to determine this risk have been extensively studied [3-12]. These risk estimates are helpful for clinical consultation and identifying high risk populations for effective treatment. Recent studies even recommended that the initiation of cardio-protective treatment for diabetic patients be based on personalized CHD risk estimates to reduce harms from overly aggressive risk factor modification [13,14]. Several equations are currently available to estimate CHD risk. Among them are algorithms derived for use in the general population such as the Systematic Coronary Risk Evaluation (SCORE) [15], QRISK [16,17], the Reynolds Risk Score for women [18] and the Framingham risk equations [4,6]. Most of these algorithms include diabetes as a risk factor when determining CHD risk. Algorithms particularly developed for use in diabetic populations include the United Kingdom Prospective Diabetes Study (UKPDS) risk equations [3] and a recent algorithm developed by Donnan et al.[10]. Among these algorithms, the UKPDS and Framingham equations [3,4] are frequently used to predict the risk of an initial CHD event for diabetic patients. Their use has also been recommended in national guidelines in various regions [19-23]. However, previous studies have shown that both the UKPDS and Framingham risk equations can give unreliable risk estimates of an initial CHD event for diabetic patients in some European and Australian cohorts [7,9,11,24,25]. Given the variation in practice patterns, patient racial/ethnic composition between these regions and the U.S., as well as changes in the standards of clinical care for patients with diabetes over the last few decades, it is not clear whether these equations can provide reliable risk estimates for adults with long-standing diabetes in the U.S. Recent estimates showed that approximately 785,000 Americans will have a new coronary event each year, with approximately 470,000 of them a recurrent event [26]. It is of both clinical and public health importance to understand and re-evaluate the risk stratifications for patients with long-standing type 2 diabetes.

In this paper, we evaluate the performance of the UKPDS [3] and Framingham initial CHD risk equations [4] in predicting CHD occurrence for adults with long-standing type 2 diabetes without an established CHD history. We also evaluated the performance of the secondary Framingham risk equation [27] in predicting CHD events among those with an established CHD history as this has not been studied among adults who have diabetes. Because adults with long-standing diabetes are known to have higher CHD risk than those without diabetes or newly diagnosed with diabetes, we focused on estimates of short-term CHD event risk using data from the Translating Research Into Action for Diabetes (TRIAD) study, a large multi-center, population-based, prospective study of diabetic adults [28].

## Methods

The TRIAD study has been described in detail elsewhere [28]. In brief, the initial TRIAD cohort consisted of 11927 community-dwelling adults with diabetes ages 18 years and older, and continuously enrolled between July 2000 and August 2001 in one of 10 managed care plans in 7 states. TRIAD data included patient surveys, medical record reviews, health plan administrative claims (inpatient, outpatient and emergency room claims), and National Death Index (NDI) data. In our analyses, we limited our sample to the 8820 TRIAD participants for whom we had medical record data 18 months prior to the baseline survey. In addition, we excluded those whose age at diabetes diagnosis was less than 30 years and were treated with insulin only at the time of the baseline survey since it is likely they had type 1 diabetes. Institutional review boards at each participating site approved the study and all participants provided informed consent.

Risk predictors such as patient age, diabetes duration (years since diagnosis), and smoking status were obtained from survey responses. Hemoglobin A1C, systolic blood pressure, diastolic blood pressure, total cholesterol, HDL, and LDL were obtained from medical records, and only the most recent values within the 18 months prior to the baseline survey were used. Patients were defined as having a CHD history if at least one of the following conditions was documented in their medical records within 3 years prior to the baseline survey: angina, MI, coronary heart disease, coronary artery disease, coronary angioplasty or coronary bypass. We also obtained additional baseline information on diabetes treatment from patient surveys, and determined the use of hypertension medication, statins and co-morbid conditions (measured by the Charlson’s score [29,30]) from medical records from 5 out of the original 6 research centers where the data were available to us.

### Outcome variables

For evaluation of each risk equation, we used the CHD event definition used in the study that provided the equations. To evaluate the UKPDS risk equations, we defined a CHD event as: a fatal or nonfatal MI (ICD-9-CM code of 410.xx administrative data; ICD-10 of I21-I22 NDI data); to evaluate the Framingham risk equations, we defined a CHD event as: angina pectoris, MI, coronary insufficiency, sudden death, or CHD death (ICD-9-CM code of 410.xx, 413, 411.89, 414.8 administrative data; ICD-10 of I20-I22 and I46.1 NDI data). For each CHD event, we calculated the “CHD event time” as the time from the TRIAD baseline survey to the first CHD event. Observations were censored at the end of 2003, date of death from another cause, or the date of the first health plan enrollment gap of more than two months, whichever came first.

### UKPDS and Framingham CHD risk equations

We evaluated various versions of UKPDS, Framingham initial and Framingham secondary CHD risk equations (Table 1). Specifically, two UKPDS risk equations were evaluated: The first equation predicts the risk of an initial CHD event for a patient with newly diagnosed diabetes and we referred to it as the *incident UKPDS risk equation*. The second equation takes into account diabetes duration when predicting the risk of an initial CHD event and we thus referred to it as the *duration UKPDS risk equation*. Framingham risk equations are gender-specific and were thus evaluated separately for men and women. We evaluated the risk equations for predicting an initial CHD event using total cholesterol categories and referred to it as *Framingham-initial*. We also evaluated the performance of the equations for predicting a subsequent CHD event and referred to it as the *Framingham-secondary*.

**Table 1:** UKPDS and framingham risk equations

**Equations**		**Formula for t-year CHD risk**
UKPDS Incident Stevens et al. [3]		$1-\exp\left\{-q\left(1-d^{t}\right)/1-d\right\}\text{,}$*where d = 1.078 and log*_*e*_*q = −4.4918+ 0.0573× (age-55)*−*0.6444× female-0.9416× Afro-Caribbean + 0.3001× smoking + 0.1681× (HbA1c-6.72) +0.0843× (SPB-135.7)/10 + 1.3468× {log*_*e*_*(TC*^*a*^*/HDL)-1.59}.*
UKPDS Duration		$1-\exp\left\{-qd^{T}\left(1-d^{t}\right)/1-d\right\}\text{,}$*where T = diabetes duration in years, and d and q were defined as above.*
Framingham-Initial Table 6 in Wilson et al. [4]	Male	$1-S_{0}{\left(t\right)}^{\exp\left(m\right)}\text{,}$*where m = 0.0483×Age-0.6595× (TC*^*a*^ *< 160 mg/dL) + 0.1769×(TC 200-239 mg/dL) + 0.5054×(TC 240-279 mg/dL) + 0.6571×(TC> = 280 mg/dL) + 0.4974×(HDL < 35 mg/dL) + 0.2431× (HDL 35-44 mg/dL)-0.0511× (HDL 50-59 mg/dL)-0.4866× (HDL > =60 mg/dL)-0.0023×(BP Optimal) + 0.2832× (BP High-normal) + 0.5217×(BP Stage-I-hypertension) + 0.6186×(BP Stage-II-IV-hypertension) + 0.4284×Diabetes + 0.5234× (Smoker)-3.0975*
		*S*_*0*_*(1)*^*b*^*=0.9946, S*_*0*_*(2) = 0.9850, S*_*0*_*(3) = 0.9770, S*_*0*_*(4) = 0.9622, S*_*0*_*(5) = 0.95592.*
	Female	$1-S_{0}{\left(t\right)}^{\exp\left(m\right)}\text{,}$*where m = 0.3377×Age-0.0027×age*^*2*^*-0.2614×(TC*^*a*^ *< 160 mg/dL) + 0.2077×(TC 200-239 mg/dL)+0.2439×(TC 240-279 mg/dL) + 0.5351×(TC > =280 mg/dL)+0.8431×(HDL < 35 mg/dL) + 0.3780×(HDL 35-44 mg/dL)+0.1979×(HDL 45-49 mg/dL)-0.4295×(HDL > =60 mg/dL)*−*0.5336×(BP Optimal)-0.0677×(BP High-normal)+0.2629×(BP Stage-I-hypertension) + 0.4657×(BPStage-II-IV-hypertension) + 0.5963×Diabetes + 0.2925×(Smoker)-9.9255*
		*S*_*0*_*(1)*^*b*^*=0.9984, S*_*0*_*(2) = 0.9933, S*_*0*_*(3) = 0.9909, S*_*0*_*(4) = 0.9858, S*_*0*_*(5) = 0.98297.*
Framingham-Secondary	Male	$1-\exp\left(-\exp\left\{\left[\log_{e}\left(t\right)-m\right]/0.9994\right\}\right)\text{,}$*where m = 4.995–0.0145×age–0.6738× log*_*e*_*(TC*^*a*^*/HDL)-0.3042×Diabetes.*
D’Agostino et al. [27]		
	Female	$1-\exp\left(-\exp\left\{\left[\log_{e}\left(t\right)-m\right]/1.0313\right\}\right)\text{,}$*where m = 13.537-0.0225×age*−*0.834×log*_*e*_*(TC*^*a*^*/HDL)-1.3713×ln(SBP)-0.7829×Diabetes-0.3669×smoker.*

^a^*TC* = Total Cholesterol.

^b^*S*_*0*_*(t)* represents the estimate of the *t*-year baseline survival rate, provided by Framingham investigators, for *t = 1,2,…,5* years.

### Risk score calculation and statistical methods

For each eligible participant, we calculated the absolute risk of a CHD event using each equation. Because the racial/ethnic composition of the TRIAD cohort differed from that in the UKPDS cohort, we used the “Afro-Caribbean” risk adjustment for African American patients and the “Caucasian or Asian-Indian” calculation adjustment for the remaining participants. Framingham risk equations were not adjusted for race/ethnicity. Because the Framingham-initial equations were published with the 10-year baseline survival rates, we obtained the 1–5 year baseline survival rates directly from the Framingham investigators.

We evaluated the risk equations for 1) how well they separate individuals who develop a CHD event from those who do not (discrimination) and 2) how close predicted risks are to observed risks [6,31] (calibration, or goodness-of-fit (GOF)). When we examined the performance of the UKPDS and Framingham-initial CHD equations, we only included patients without a CHD history; when we examined the performance of the Framingham-secondary equations, we only included patients with a CHD history.

Discrimination was evaluated using the Harrell’s c-index for censored data (R package *HMISC* available on CRAN at http://cran.r-project.org), a statistic similar to the area under a receiver operating characteristic curve [32]. In general, a c-index greater than 0.7 indicates good discrimination while a value of 0.5 indicates discrimination equivalent to chance. Intermediate values indicate limited discriminating utility. Calibration plots were generated and Hosmer-Lemeshow-type chi-square statistics [6,33] were calculated to compare differences between predicted and observed risks based on deciles of risk scores. We conservatively defined lack of calibration as chi-square values greater than 23.2 (the 99^th^ percentile of chi-square distribution with 10 degrees of freedom). We also recalibrated the UKPDS and Framingham risk equations by replacing the average values of predictors and event rates in the original populations by those in the TRIAD population. Specifically, we used the method of D’Agostino et al.[6] to recalibrate the Framingham-initial equations and the method of van Houwelingen [34] to recalibrate the UKPDS and Framingham-secondary equations because the latter were parametric models.

To investigate the difference between study populations with regard to the effect of risk predictors, we fitted each of these equations on TRIAD data and compared the estimates of relative risk (hazard ratio) using the method described in D’Agostino et al. [6]. Specifically, we fitted the Cox regression models and used the same CHD event definition as well as the risk predictors in the original equations. For simplicity, the models using the TRIAD data were all referred to as the TRIAD models. Regression coefficients, hazard ratio (HR) estimates, Harrell’s c-index and GOF statistics [35] were calculated.

Missing data ranged from 1.3% (smoking) to 20.7% (HDL), and was handled in the data analysis using multiple imputation. Imputations were generated using a sequential regression imputation method via the software package IVEware, and results were combined using Rubin’s rule implemented in SAS v9.2 MIANALYZE procedure [36-38].

## Results and discussion

### Results

The follow-up time ranged from 0–3.5 years with a median of 2.7 years (inter-quartile range = 0.9). In the overall TRIAD sample (n = 8303 subjects), there were 319 UKPDS-defined and 592 Framingham-defined CHD events. Among those without a history of CHD (n = 5914), there were 120 UKPDS-defined and 218 Framingham-defined CHD events over the analysis period, with a corresponding 3.5-year CHD event rate (Kaplan-Meier estimate) of 3.0% (95%CI: 2.3%, 3.8%) and 5.1% (95%CI: 4.2%, 6.0%), respectively. Mean age at baseline was 59.8 (SD = 12.3) years with a mean diabetes duration of 10.6 (SD = 9.1) years; 56.7% were female, 39.7% were non-Hispanic White, and nearly 18% were smokers. Of those for whom we had information on medication use and co-morbid burden (n = 4602), 78.4% took oral medication, 26.4% took insulin, 68.7% took hypertension medication, 28% took statins, and 50% had a Charlson score > =2. For those with a prior CHD history (n = 2389), there were 199 UKPDS-defined and 374 Framingham-defined CHD events, with a corresponding 3.5-year CHD event rate of 11.9% (95%CI: 9.6%, 14.2%) and 20.9% (95%CI: 17.9%, 23.9%), respectively. Compared to those without a CHD history, they were generally older with longer diabetes duration and better cholesterol control; they also took more hypertension medication, statins, and suffered from a greater number of comorbid conditions (Table 2).

**Table 2:** Demographics and clinical characteristics of TRIAD participants

	**Without CHD History**	**With CHD History**
N	5914	2389
Age at baseline survey (SD) (years)	59.8 (12.3)	66.1 (10.4)
Female (%)	56.7	44.9
Race/Ethnicity (%)		
Non- Hispanic white	39.7	51.0
Non- Hispanic black	17.3	15.9
Hispanic	17.7	14.4
Hawaiian/Pacific Islander	16.6	10.5
Other	8.8	8.2
Duration (SD) (years)	10.6 (9.1)	13.6 (10.2)
History of CHD event^a^ (%)	0.0	100.0
HbA1C (SD)	8(1.9)	7.9(1.8)
Total Cholesterol (SD) (mg/dL)	200.8 (42.1)	191.9 (45.5)
HDL Cholesterol (SD) (mg/dL)	47.5 (12.9)	44.7 (12.8)
LDL Cholesterol (SD) (mg/dL)	116.1 (34.9)	108.8 (36.7)
Systolic Blood Pressure (SD) (mmHg)	136.4 (18.4)	136.7 (19.8)
Smoker (%)	18.5	17.5
Diabetes Treatment^b^ (%)		
Diet only	7.6	6.2
Oral medication	66.0	57.5
Insulin	14.0	20.1
Insulin and oral medication	12.4	15.4
Other medication^b^ (%)		
Hypertension	68.7	88.5
Statin	28.0	54.6
Co-morbidity Charlson’s score^b^ (%)		
<1	5.2	1.9
> = 1 to 2	44.0	17.4
> = 2 to 3	28.9	24.1
> = 3	21.9	54.6
Number of incident UKPDS CHD events^c^	120	199
Number of incident Framingham CHD events^d^	218	374
3.5-year UKPDS CHD event rate (95%CI)	3.0%	11.9%
	(2.3%, 3.8%)	(9.6%, 14.2%)
3.5-year Framingham CHD event rate (95%CI)	5.1%	20.9%
	(4.2%, 6.0%)	(17.9%, 23.9%)

^a^CHD history was identified in the medical record documentation, if at least one of the following conditions occurred in medical records 3 years prior to the baseline survey: angina, MI, other coronary heart disease or coronary artery disease, coronary angioplasty or bypass.

^b^Data were obtained from 4602 patients without a CHD history and 2029 patients with a CHD history.

^c^UKPDS CHD event is defined as fatal or non-fatal MI.

^d^Framingham CHD event is defined as angina pectoris, MI, coronary insufficiency, sudden or non-sudden CHD death.

**Figure 1:**
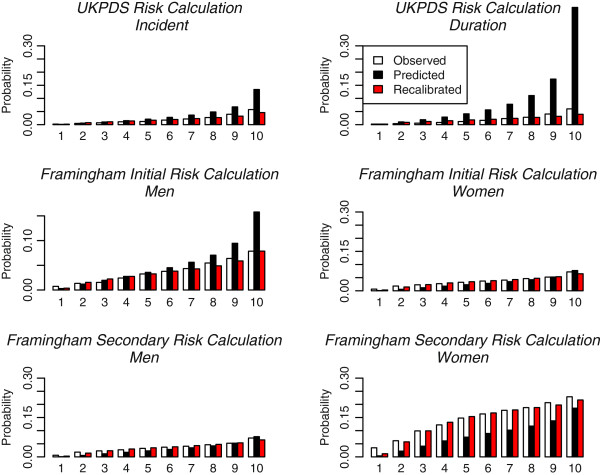
**Calibration plot of UKPDS and Framingham CHD risk equations.** X-axes refer to deciles of predicted risk scores using the UKPDS and Framingham CHD risk equations, where appropriate. Each bar in the graph represents the average of the observed and predicted risk scores from the UKPDS and Framingham risk equations.

Evaluation of discrimination and calibration of the UKPDS and Framingham risk equations is summarized in Table 3. The Harrell’s c-index of discrimination for the UKPDS risk equations was generally low: 0.63 (95%CI: 0.58, 0.68) for the incident equation and 0.64 (95%CI: 0.59, 0.69) for the duration equation. The (unadjusted) GOF chi-square value for both UKPDS equations exceeded the established cutoff, indicating a lack of calibration. Specifically, these equations tended to over-estimate CHD risk (Figure1). Similar results were found in our evaluation of Framingham-initial and Framingham-secondary risk equations. Specifically, we found that discrimination was generally low, and so was the calibration except the Framingham-initial equation for women and the Framingham-secondary equation for men. When using these equations, risk tended to be over-estimated for men without a CHD history, and under-estimated for women with a CHD history. After recalibration, the goodness-of-fit of all of the risk equations greatly improved as indicated by the adjusted GOF chi-square statistics that are below the cutoff values (Table 3 and Figure1).

**Table 3:** Discrimination and calibration of UKPDS and Framingham CHD Risk Equations

		**N (Number of CHD events)**	**Discrimination Harrell’s c-index (95%CI)**	**Calibration GOF Chi-Square Statistics**	
				**Unadjusted**^**a**^	**Adjusted**^**a**^
UKPDS	Incident	5914 (120)	0.63 (0.58, 0.68)	55.64	2.18
	Duration	5914 (120)	0.64 (0.59, 0.69)	446.73	7.65
Framingham-Initial	Men	2560 (97)	0.61 (0.55, 0.67)	24.28	0.88
	Women	3354 (121)	0.59 (0.54, 0.64)	17.27	5.85
Framingham-Secondary	Men	1317 (209)	0.55 (0.51, 0.59)	19.45	8.52
	Women	1072 (165)	0.54 (0.49, 0.58)	77.59	5.23

^a^Unadjusted and adjusted refer to the GOF chi-square statistics before vs. after recalibration, respectively.

For the TRIAD version of the UKPDS equations (Table 4), only age and systolic blood pressure remained significantly associated with CHD (p<0.05). HRs associated with age, gender, racial difference, log_e_(total cholesterol/HDL) and diabetes duration were significantly different, mostly smaller, than those in the original equations (p<0.05). For the TRIAD versions of Framingham initial and secondary equations, almost none of the risk predictors were significantly associated with CHD. Most HR estimates did not significantly differ from those from the original estimates, except for age and HDL for women in the initial equation. However, because the standard errors of regression coefficient estimates for the original Framingham-initial equations were not provided in Wilson et al. [4], thus they were not included in these calculations and the actual p-value might be slightly bigger. The goodness-of-fit of these TRIAD models is generally good (p<0.05), but the discriminating power was low (all the c-indexes were < 0.65).

**Table 4:** Regression coefficients, hazard ratio (HR), and goodness-of-fit of TRIAD Models and comparisons of the hazard ratios with the original risk equations

		**TRIAD models**				**Original**
**Model**	**Variable**	**Coeff**	**Standard Error or 95%CI**	**p-value**	**HR**	**HR**
Incident	Age (year)	0.038	0.008	<.0001	1.038*	1.059
UKPDS	Female (yes/no)	−0.109	0.188	0.561	0.896*	0.525
	African American (yes/no)	−0.154	0.281	0.585	0.858*	0.390
	(yes/no)	0.440	0.235	0.061	1.552	1.350
	HbA1c	0.075	0.054	0.170	1.077	1.183
	SBP	0.010	0.005	0.034	1.010	1.008
	log_e_ (TC^a^/HDL)	0.588	0.359	0.102	1.801*	3.845
	C index	0.66	(0.61, 0.71)			
	p-value for GOF	0.48				
UKPDS	Age (year)	0.035	0.009	<.0001	1.035*	1.059
	Female (yes/no)	−0.122	0.188	0.517	0.885*	0.525
	African American (yes/no)	−0.160	0.281	0.569	0.852*	0.390
	Smoker (yes/no)	0.450	0.235	0.055	1.568	1.350
	A1c	0.066	0.055	0.234	1.068	1.183
	SBP	0.010	0.005	0.037	1.010	1.008
	log_e_ (TC^a^/HDL)	0.612	0.358	0.089	1.844	3.845
	Duration (year)	0.012	0.009	0.177	1.012*	1.078
	C index	0.66	(0.61, 0.71)			
	p-value for GOF	0.62				
Framingham						
-Initial	Age (year)	0.037	0.009	<.0001	1.038	1.049
Male	TC, mg/dL					
	<160	−0.154	0.385	0.690	0.857	0.517
	160-199	-	-	-	-	-
	200–239	0.415	0.239	0.083	1.514	1.194
	240-279	0.052	0.422	0.903	1.053	1.658
	≥280	−0.228	1.055	0.829	0.796	1.929
	HDL, mg/dL					
	<35	−0.004	0.355	0.990	0.996	1.645
	35-44	−0.179	0.306	0.560	0.836	1.275
	45-59	-	-	-	-	-
	50-59	−0.297	0.397	0.455	0.743	0.950
	≥60	−0.272	0.465	0.558	0.762	0.615
	Blood Pressure^b^					
	Optimal	−0.513	0.459	0.264	0.599	0.998
	Normal	-	-	-	-	-
	High normal	−0.059	0.326	0.857	0.943	1.327
	Hypertension stage I	0.037	0.299	0.903	1.037	1.685
	Hypertension stage II–IV	−0.009	0.376	0.982	0.991	1.856
	Smoker	0.216	0.273	0.429	1.241	1.688
	C index	0.65	(0.59, 0.70)			
	p-value for GOF	0.53				
Framingham						
-Initial	Age (year)	0.157	0.080	0.050	1.170*	1.402
Female	Age^2^ (year)	−0.001	0.001	0.103	0.999	0.997
	TC, mg/dL					
	<160	−0.759	0.547	0.168	0.468	0.77
	160-199	-	-	-	-	-
	200–239	−0.072	0.233	0.756	0.930	1.231
	240-279	0.302	0.302	0.318	1.353	1.276
	≥280	0.693	0.356	0.052	1.999	1.708
	HDL, mg/dL					
	<35	0.415	0.461	0.369	1.515	2.324
	35-44	0.132	0.291	0.652	1.141	1.459
	50-59	0.210	0.336	0.534	1.233	1.219
	50-59	-	-	-	-	-
	≥60	0.372	0.269	0.167	1.451**	0.651
	Blood Pressure^b^					
	Optimal	−0.638	0.469	0.174	0.528	0.586
	Normal	-	-	-	-	-
	High normal	−0.298	0.318	0.348	0.742	0.935
	Hypertension stage I	0.119	0.270	0.660	1.126	1.301
	Hypertension stage II–IV	0.340	0.301	0.259	1.404	1.593
	Smoker	0.440	0.236	0.062	1.553	1.340
	C index	0.67	(0.63, 0.72)			
	p-value for GOF	0.62				
-Secondary	Age (year)	0.006	0.007	0.426	1.005	1.015
Male	log_e_(TC^†^/HDL)	0.604	0.261	0.023	1.827	1.962
	C index	0.55	(0.51, 0.59)			
	p-value for GOF	0.27				
-Secondary	Age (year)	0.012	0.008	0.131	1.012	1.022
Female	log_e_ (TC^†^/HDL)	0.336	0.335	0.319	1.395	2.245
	log_e_ (SBP)	0.793	0.560	0.157	2.248	3.780
	Smoker (yes/no)	−0.266	0.240	0.269	0.764	1.427
	C index	0.55	(0.50, 0.59)			
	p-value for GOF	0.23				

^a^TC = Total Cholesterol.

^b^Blood Pressure categories (mmHg): Optimal: Systolic < 120, Diastolic <80; Normal: Systolic 120–129, Diastolic <80-84; High normal: Systolic 130–139, Diastolic 85–89; Hypertension stage I: Systolic 140–159, Diastolic 90–99, Hypertension stage II–IV: Systolic ≥ 160, Diastolic ≥100 [4].

*p<0.05, **p<0.01, ***P<0.001 in the HR comparisons of the TRIAD models with original models. Note that in comparing HRs with the Framingham-initial equations, the actual p-values may be slightly bigger than those reported here because the standard errors of the original regression coefficient estimates were not provided in original study [4] and they were not included in these calculations.

### Discussion

Our study showed that the UKPDS and Framingham risk equations may be inappropriate for predicting short-term risk of CHD events for adults with long-standing type-2 diabetes. All of these equations exhibited low discriminating power. All except the Framingham-initial equation for women and Framingham-secondary equation for men had low levels of calibration. Our findings were similar to those found in other studies, including van Dieren et al.[9] that evaluated the performance of the UKPDS risk equations by looking at the CHD event risk at 4, 5, 6 and 8 years, and the ADVANCE trial that evaluated the 4-year CHD risk [11]. Separate analyses also showed that the UKPDS and Framingham-initial equations tended to greatly underestimate the risk of a CHD event for patients with a CHD history and discriminating power was consistently low (data not shown).

Several factors may explain our findings of low discrimination and calibration of these equations. First, our study participants had an average of diabetes duration longer than ten years and were community-based health plan enrollees. In contrast, the UKPDS cohort was derived from a clinical trial that only included individuals newly diagnosed with diabetes, while the Framingham cohort only included a small proportion of individuals with diabetes. The general health status, patterns of medication use, and presence of other CHD risk factors in the TRIAD cohort (Table 1) likely differ from those in the previous studies. For instance, at least 68% of TRIAD participants received anti-hypertensive therapy, while fewer than 30% of UKPDS participants and fewer than 10% of Framingham participants had received anti-hypertensive therapy [4,39]. Moreover, the participants in TRIAD were on average 10 years older than those in the UKPDS and Framingham cohorts [3,4]. Risk estimates of these equations may need adjustment when applied to older patients because older patients generally are more susceptible to cardiovascular risks, such as higher blood pressure and declining levels of physical activity. They also tend to have more co-morbid conditions, both physically and mentally; some co-morbid conditions may even lead to non-cardiovascular deaths as competing risks [40-42]. In addition, the racial/ethnic composition of our cohort included greater numbers of non-white patients than the UKPDS or Framingham cohorts. Risk profiles of minority groups not specified by the algorithms (e.g., Hispanics and Asians other than South Asians) may be significantly different. Finally, UKPDS and Framingham risk equations were developed from cohorts formed between the 1970s and the 1990s [3,4]. Treatment of type 2 diabetes and management of cardiovascular risk among diabetic adults have improved substantially since then [43-46].

In investigating the relations of these risk equations with the risk of CHD events in the TRIAD cohort, we found that most predictors were not statistically significant in the TRIAD models. This may be caused by patterns of medication use (e.g., hypertensive drugs, diabetes treatment and statins; Table 1), comorbid conditions, or other factors unaccounted for in the risk equations. Some recent algorithms do include medication as a predictor, such as the prediction model of Donnan et al. [10]. To improve the discriminating power of these models, additional variables, such as medications, family history, life-style related risk factors, socioeconomic status, co-morbid conditions, and novel biomarkers [20,26,45,47,48], may need to be incorporated in the risk equations.

Recent studies suggest that using a “blanket” approach or aggressive risk factor modification (e.g., lowering LDL and/or blood pressure), based on the public notion that diabetes is a CHD risk equivalent, may lead to an overly aggressive treatment and thus offset a patient’s net benefit from treatment [13,14,49]. These authors instead recommend that patients be treated based on appropriate personalized CHD risk estimates. Our analysis showed that the UKPDS and the Framingham-initial equation for men tend to overestimate the initial CHD risk for diabetic patients in a contemporary cohort. Refinement of these equations to reflect the CHD prognostics in a modern diabetic cohort are needed to provide reliable risk estimates to inform effective treatment.

The strength of our study is the large sample size. However, our study has some limitations. The longest follow-up time for CHD events in our study was 3.5 years, thus limiting our ability to evaluate the use of these equations to predict longer-term CHD event risk. With longer follow-up (e.g., 10 years), it is possible that these equations may provide better predictions of CHD risks. However, our study population is more susceptible to CHD than the general population, and the average age of our study population tends to be older (> = 60 years). Evaluating short-term CHD risk in this population can provide useful insights for disease management and treatment. Since CHD events were identified mostly through health plan administrative data, identification of these events may not be complete [50], particularly for patients with “silent” infarction who do not seek health care and thus are not represented in claims data. As a consequence, while the numbers of CHD events represent what a health plan should expect from a typical diabetic patient population, it will miss events that are not clinically recognized.

## Conclusion

Our study shows that UKPDS and Framingham CHD risk equations may have limited utility to predict CHD risk for adults with long-standing type-2 diabetes in a U.S. population. It is of both clinical and public health importance to understand the risk levels, risk factors, effective treatment and prevention of the occurrence of a CHD event. Evaluation of these commonly used risk equations for predicting short-term risk of CHD events in this cohort is important in that risk-stratification is frequently used for clinical decision-making, and use of these risk equations are likely to give unreliable risk estimates. In addition, given the high rates of CHD and recurrent CHD events in adults with diabetes, refinement of these risk equations may help to identify high-risk populations that can benefit from public health approaches to risk reduction. The number of adults with long-standing diabetes and associated CHD in the U.S. is high [26,51]. Our findings highlight the need for new or more refined CHD risk equations to re-assess the CHD event risk and understand factors that influence CHD event risk in adults with prevalent diabetes in a modern U.S. cohort.

## Competing interests

The authors declare that they have no competing interests.

## Authors’ contributions

SEL contributed to the conception, design, analysis, interpretation of data, and wrote the manuscript; GLB contributed to the conception, design, interpretation of data and revised manuscript; JCC contributed to interpretation of data and revised manuscript; DB contributed to the data analysis, and revised manuscript; AJK contributed to the interpretation of data, and revised manuscript; RBG contributed to the data analysis and revised manuscript, YL contributed to the statistical analysis and interpretation of data; SVR contributed to the conception and revised manuscript; LNM contributed to the interpretation of data and revised manuscript; BW contributed to the interpretation of data and revised manuscript; DM contributed to the interpretation of data and revised manuscript; AFB contributed to the conception, design, interpretation of data and revised manuscript. All authors contributed to final approval of the version to be published.

## Pre-publication history

The pre-publication history for this paper can be accessed here:

http://www.biomedcentral.com/1472-6823/12/12/prepub
